# Partially Hydrolysed Whey Has Superior Allergy Preventive Capacity Compared to Intact Whey Regardless of Amoxicillin Administration in Brown Norway Rats

**DOI:** 10.3389/fimmu.2021.705543

**Published:** 2021-08-31

**Authors:** Katrine Bækby Graversen, Jeppe Madura Larsen, Signe Schultz Pedersen, Laila Vestergaard Sørensen, Heidi Frahm Christoffersen, Lotte Neergaard Jacobsen, Susanne Halken, Tine Rask Licht, Martin Iain Bahl, Katrine Lindholm Bøgh

**Affiliations:** ^1^National Food Institute, Technical University of Denmark, Kgs. Lyngby, Denmark; ^2^Research & Development, Arla Foods Ingredients Group P/S, Videbæk, Denmark; ^3^Strategic Business Unit Pediatric, Arla Foods Ingredients Group P/S, Viby J, Denmark; ^4^Hans Christian Andersen Children’s Hospital, Odense University Hospital, Odense, Denmark

**Keywords:** food allergy, cow’s milk allergy, hypoallergenic infant formula, allergy prevention, β-lactam antibiotic, microbiota, IgA, iTreg

## Abstract

**Background:**

It remains largely unknown how physicochemical properties of hydrolysed infant formulas influence their allergy preventive capacity, and results from clinical and animal studies comparing the preventive capacity of hydrolysed infant formula with conventional infant formula are inconclusive. Thus, the use of hydrolysed infant formula for allergy prevention in atopy-prone infants is highly debated. Furthermore, knowledge on how gut microbiota influences allergy prevention remains scarce.

**Objective:**

To gain knowledge on (1) how physicochemical properties of hydrolysed whey products influence the allergy preventive capacity, (2) whether host microbiota disturbance influences allergy prevention, and (3) to what extent hydrolysed whey products influence gut microbiota composition.

**Methods:**

The preventive capacity of four different *ad libitum* administered whey products was investigated in Brown Norway rats with either a conventional or an amoxicillin-disturbed gut microbiota. The preventive capacity of products was evaluated as the capacity to reduce whey-specific sensitisation and allergic reactions to intact whey after intraperitoneal post-immunisations with intact whey. Additionally, the direct effect of the whey products on the growth of gut bacteria derived from healthy human infant donors was evaluated by *in vitro* incubation.

**Results:**

Two partially hydrolysed whey products with different physicochemical characteristics were found to be superior in preventing whey-specific sensitisation compared to intact and extensively hydrolysed whey products. Daily oral amoxicillin administration, initiated one week prior to intervention with whey products, disturbed the gut microbiota but did not impair the prevention of whey-specific sensitisation. The *in vitro* incubation of infant faecal samples with whey products indicated that partially hydrolysed whey products might confer a selective advantage to enterococci.

**Conclusions:**

Our results support the use of partially hydrolysed whey products for prevention of cow’s milk allergy in atopy-predisposed infants regardless of their microbiota status. However, possible direct effects of partially hydrolysed whey products on gut microbiota composition warrants further investigation.

## Introduction

Cow’s milk allergy (CMA) is the most common food allergy in infants and young children with a prevalence of 0.5-3% (1–3). Non-exclusively breastfed infants suffering from CMA are recommended the use of hypoallergenic infant formula (IF), most often based on extensively hydrolysed cow’s milk proteins (4). Enzymatic hydrolysis degrades proteins into peptide fragments and free amino acids (AA) and thereby eliminates the antibody-binding epitopes, thus reducing the risk of inducing an allergic reaction. Hypoallergenic IFs based on hydrolysed cow’s milk proteins are divided into extensively (eHF) and partially (pHF) hydrolysed IFs based on the size distribution of the peptides. There is currently no consensus for a definition of eHF and pHF. The American Academy of Pediatrics has suggested a definition for eHF as containing only peptides with a molecular weight below 3 kDa (corresponding to approx. 27 AAs), and pHF as those that mostly consists of oligopeptides with a molecular weight below 5 kDa (corresponding to approx. 45 AAs) (5).

The recommendation for prevention of CMA and other allergic diseases in non-exclusively breastfed atopy-prone infants is highly debated. Two recent systematic reviews concluded that there is not substantial evidence that hydrolysed IFs prevent CMA nor any other allergic diseases (6, 7). The European guidelines currently recommends the use of hydrolysed IFs for food allergy prevention in high-risk infants below the age of 4 months (8), while the American (9) and Australian-Asian (10) guidelines were recently updated to no longer recommend hydrolysed IFs for prevention of CMA or other allergic diseases.

Few clinical studies have evaluated CMA as an endpoint when comparing the effect of pHF to conventional IF in high-risk infants (11–13), and results from these studies differ and are not clearly in favour of pHF. However, as discussed in the recent Cochrane review (7), the study designs are problematic. Animal studies indicate that the allergenicity of pHF is reduced compared to conventional IF (14, 15), but that the preventive capacity may also be reduced (16–18), or at best similar to conventional IF (19, 20).

Variation between studies may be due to the use of different IFs with different physicochemical characteristics. Classification definitions are ambiguous, and high variation in physicochemical profiles (21) and peptide sequence signatures (22) have indeed been reported among different commercially available hydrolysed IFs from the same protein source and within the same classification. This implies that different IFs may have different immunological effects.

Changes in environmental factors and lifestyle has been associated with the increased prevalence in food allergy observed in the last decades (23), likely related to alterations in the gut microbiota. Epidemiological studies suggest an association between antibiotic exposure in early life and CMA (24–26). Recent studies have further indicated a direct causal relationship between gut microbiota and the development of CMA in murine models (27, 28). However, knowledge on how environmental factors, including antibiotic exposure, influence allergy prevention remains scarce.

In the current study, amoxicillin was used to manipulate the microbiota composition during intervention with whey products in Brown Norway (BN) rats, as amoxicillin is the most widely used β-lactam antibiotic in Europe (29), and is frequently prescribed for treatment of paediatric infections (30). Prevention of CMA was achieved by *ad libitum* administration of intact and hydrolysed whey products with different physicochemical properties to cow’s milk naïve BN rats. Preventive capacity was evaluated as the capacity to prevent whey-specific sensitisation and allergic reactions to intact whey after intraperitoneal (ip) post-immunisations with intact whey (31). BN rats are high-IgE responders resembling atopy-prone individuals in their predisposition to develop food allergy (20, 32). To our knowledge, this is the first study to show that hydrolysed whey products are superior to an intact whey product for preventing whey-specific sensitisation.

## Materials and Methods

### Product Characterisation

Four protein ingredients for IF were included in the study: One intact whey product (iW), two non-filtered, partially hydrolysed whey products, and one filtered extensively hydrolysed whey product (eHW) with degree of hydrolysis of 7.2%, 22.4% and 27%, respectively. All products were made from whey protein concentrate, with hydrolysates produced in a one-step hydrolysis with commercially available food grade enzymes which were heat inactivated upon termination of hydrolysis. The eHW was filtered by ultrafiltration in order to remove larger peptides and aggregates hereof. All products were kindly provided by Arla Foods Ingredients. The products were characterised in terms of degree of hydrolysis, AA composition, peptide size distribution and protein aggregation (methods are described in **Supplementary Material S1**).

### Animals

BN rats from our in-house breeding colony (National Food Institute, Technical University of Denmark, Denmark) were housed in Makrolon cages and kept at a 12 h light:dark cycle, at a temperature of 22 ± 1°C and a relative humidity of 55 ± 5%. Rats were observed twice daily and clinical signs recorded. The rats were fed a milk-free diet for ≥10 generations. The diet was produced in-house and based on rice flour, potato protein and fish meal as protein sources, as previously described (33), with the exception of maize flakes being substituted with rice flour. Ethical approval was given by the Danish Animal Experiments Inspectorate (2015-15-0201-00553-C1). The experiments were overseen by the National Food Institute’s in-house Animal Welfare Committee for animal care and use.

#### Animal Experiment

**Figure 1:**
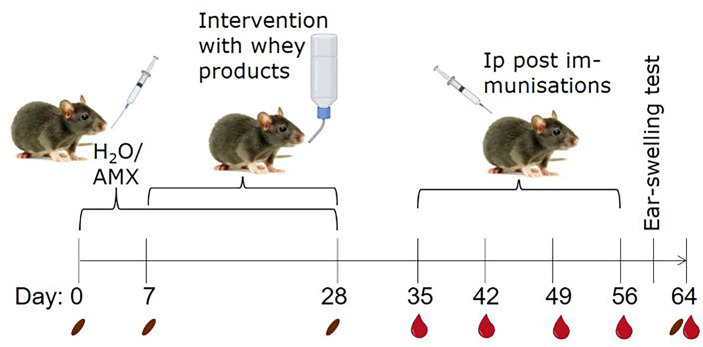
Animal experimental design. To create two groups with different microbiota compositions, rats were gavaged daily with either amoxicillin (AMX) or water (H_2_O), as conventional control, from one week before and during whey product interventions. Brown Norway rats were administered one of four different whey products in their drinking water or water alone, *ad libitum* for three weeks. Subsequently, all rats were post-immunised by weekly intraperitoneal (ip) injections with intact whey (iW) for sensitisation for four weeks. The day before euthanisation, rats were subjected to an ear-swelling test by intradermal injection of iW. Blood and faecal samples were collected throughout the study on the indicated days. The pictures originate from BioRender.com and Colourbox.

BN rats of both sexes, aged 3-7 weeks (mean and median ages of each group in **Table S1**), were allocated into 10 groups of 12 rats. Half of the groups were daily gavaged with 30 mg amoxicillin in 0.5 mL milli Q water and the other half with water alone (conventional) from Day 0-28 (**Figure 1**). From Day 7-28, one amoxicillin group and one conventional group were given one of the four whey products in their drinking water (12.5 g protein per litre water) *ad libitum* to induce tolerance, or water alone for control. The animal experiment was divided into staggered sets. Each set consisted of one conventional and one amoxicillin group that received the same whey product intervention. Water bottles with whey products were replaced daily and liquid consumption in each cage estimated. The average daily whey protein consumption per animal was 165.6 mg. There was no statistically significant difference in protein consumption between the different whey product intervention groups. One week after discontinuing administration of whey products and/or amoxicillin, all rats were post-immunised ip with 100 µg iW in 0.5 mL phosphate buffered saline (PBS; 137 mmol/L NaCl, 2.7 mmol/L KCl, 10 mmol/L Na_2_HPO_4_, 1.8 mmol/L KH_2_PO_4_, pH 7.2) once a week for four weeks (Day 35, 42, 49, and 56). Blood samples from the sublingual vein were collected after intervention with whey products (Day 28) and weekly throughout the rest of the study (always before dosing). In total, five blood samples were collected from each animal, each estimated to constitute less than 7.5% of the total blood volume. Two faecal samples per rat were collected on Days 0, 7, 28 and 64. One was stored at -80°C for microbiota analysis and one was prepared for IgA quantification. Serum and faecal water were prepared as previously described (34).

#### Ear-Swelling Test

The day before termination (Day 63), all rats were subjected to an ear-swelling test. The rats were anaesthetised with hypnorm-dormicum, and their ear thickness was measured at the same spot before and one hour after intradermal injection of 10 μg of iW in 20 μL PBS. In parallel, naïve untreated rats were subjected to the same treatment.

#### Dissection

One week after the last post-immunisation (Day 64), rats were euthanised by exsanguination using carbon dioxide inhalation as anaesthesia. To assess intestinal uptake of whey protein, rats were dosed with 100 mg of iW in 1 mL milli Q water by oral gavage 15 min prior to exsanguination as previously described (34). The dissection, storage and preparation of tissues were previously described in details (34). In brief, intestinal content from the proximal small intestine was stored at -80°C for microbiota analysis. Small intestinal epithelium and lamina propria (LP) were stored at -80°C for total protein extraction and β-lactoglobulin quantification as previously described (34). A piece of the small intestine and the colon was fixed in 4% (w/v) paraformaldehyde for histology as previously described (34).

### Flow Cytometry

Single cells were prepared from mesenteric lymph nodes (mLN), small intestinal Peyer’s patches (PP) and small intestine LP, and along with whole blood cells stained for flow cytometry analysis of different lymphocyte populations essentially as previously described (34). In brief, after 5 min incubation with blocking solution (10% (v/v) rat serum and 5 µg/mL anti-CD32 (BD Biosciences) in FACS buffer), cells were stained for 20 min on ice with 50 μL/10^6^ cells antibody cocktail containing 2 µg/mL of each of the following antibodies: anti-B220-PE (HIS24, BD Biosciences), anti-CD3-PerCp-Cy5 (eBioG4.18, Thermo Fisher Scientific), anti-CD4-PE-Cy7 (OX-35, BD Biosciences), anti-CD45-APC-Cy7 (OX-1, Thermo Fisher Scientific), anti-CD25-BV421 (OX-39, BD Biosciences) in FACS buffer. Whole blood was lysed by 10 min incubation at RT with 1 mL VersaLyse (Beckman Coulter, Brea, CA, US) after surface staining.

Subsequently, cells were fixed and permeabilised by BD Transcription Factor buffer set (BD Biosciences) according to the manufacture’s protocol. After 5 min incubation with blocking solution cells were stained for 40 min on ice by 50 µL/10^6^ cells of 5 µg/mL anti-Foxp3-FITC (JFK-16s, Thermo Fisher Scientific) and 2 µg/mL anti-Helios-Alexa Flour 647 (22F6, BD Biosciences). Data was acquired on BD FACSCanto II system (BD Bioscience) and analysed by FlowJo (Version 10.4.2, BD Bioscience). The gating strategy can be found in **Figure S1**.

### Quantification of Antibodies by ELISA

Both iW-specific IgG1 and IgA antibodies were quantified in serum by indirect ELISA as previously described (35, 36). iW-specific IgE antibodies were quantified in serum by antibody-capture ELISA as previously described (35, 36) with the exception that plates were initially blocked with 5% (v/v) rabbit serum (Biowest, Nuaillé, France), and iW-specific IgE was detected by 50 µL/well of 0.2 µg/mL of 10:1 digoxigenin (DIG)-coupled iW in blocking solution. Total IgA was quantified in serum and faecal water by sandwich ELISA as previously described (34).

### Amplicon Sequencing and Analysis

DNA extraction and amplicon sequencing of the 16S rRNA gene by Ion Torrent sequencing was performed essentially as previously described (34) (more details in **Supplementary Material S1**).

Raw sequence data in FASTQ format was processed using CLC bio genomic workbench (Qiagen, Hilden, Germany) in order to de-multiplex samples, remove sequencing primers, quality trim with default settings (remove low quality nucleotides pbase-calling error=0.05, trim ambiguous nucleotides=2) and discard reads with a final length outside the range 125-180bp. Further quality trimming was performed in Divisive Amplicon Denoising Algorithm 2 (DADA2, Version 1.10.1) with default settings as described elsewhere (37). Finally, an amplicon sequencing variant (ASV) table was constructed which contains the counts of each sequence variant in each sample. All sequence reads were taxonomically classified using the Ribosomal Database Project database (38).

The ASV table was imported into the “Quantitative Insights Into Microbial Ecology” (Qiime) 2 pipeline (Version 2019.1) (39), and α and β diversity metrics as well as taxonomic distributions in samples were calculated by the function “diversity core-metrics-phylogenetic” based on a rooted phylogenetic tree. Faecal samples were rarefied to 10,000 reads and small intestine samples were rarefied to 3,000 reads to eliminate bias from uneven sampling depth.

### *In Vitro* Incubation Experiment

#### Human Infant Faecal Samples

Faecal samples were obtained from three healthy human infant donors. All donors were 1-4 months old, exclusively breastfed, and had never received antibiotics. The study was approved by The Danish National Committee on Health Research Ethics (H-16030078), and written informed consent was obtained from the parents of the faecal donors. Faecal samples were collected in the participants’ homes in airtight containers and stored at 4°C until delivery to the laboratory, where they were processed immediately. All samples were processed within 12 h. Faecal samples were mixed 1:1 (w/v) with 30% glycerol in saline and frozen at -80°C in aliquots. For the incubation experiment, faecal samples were thawed, and 25% (v/v) faecal slurry was prepared by mixing the samples with anoxic sterile PBS. Faecal slurries were centrifuged at 200*g* for 3 min at 4°C and only the supernatants were used.

#### Small-Scale *In Vitro* Incubations

A small-scale *in vitro* incubation method (40) was used to assess the effect of whey products on human infant faecal-derived bacteria as well as on a defined mixed culture of *Bifidobacterium longum* ssp. infantis (NCIMB 702205), *Lactobacillus rhamnosus* (ATCC 53103) and *Enterococcus faecalis* (DSM 20478) (more information in **Supplementary Material S1**).

Each whey product was dissolved in minimal medium (**Supplementary Material S1**) to obtain a final protein concentration of 12.5 mg/mL. In 24-well plates, 80 µL of faecal slurry supernatant or defined mixed culture was inoculated in 1920 µL of whey product-containing media or non-supplemented medium controls and incubated in an anaerobic cabinet at 37°C for 24 h. Each combination was performed in triplicates. Total DNA was extracted by DNeasy PowerLyzer PowerSoil Kit (Qiagen) and the relative abundance of the genera *Bifidobacterium*, *Lactobacillus* and *Enterococcus* relative to the total bacteria was analysed by qPCR with primers previously validated for use in gut communities (41) (described in **Supplementary Material S1 **and** Table S2**). Each amplification reaction was performed in triplicate for each incubation sample.

### Statistics

Graphs and statistical analyses were generated in Prism version 8.1.1 (GraphPad, San Diego, CA, US). ELISA results were expressed as log2 antibody titres. Normal distribution of data was tested by D’Agostino-Pearson normality test. For data that passed the normality test, group-means are indicated on graphs and differences between groups were analysed by t-test (two groups) or one-way ANOVA followed by Tukey’s post-test for multiple comparison. For data that did not pass the normality test, group-medians are indicated on graphs, and group differences were analysed by non-parametric Mann-Whitney test (two groups) or Kruskal-Wallis test followed by Dunn’s post-test for multiple comparison.

Differences between β diversity of groups were assessed by applying ANalysis Of SImilarities ANOSIM (42) to weighted UniFrac distances in Qiime 2. Differential abundant genera between the amoxicillin and conventional groups were determined by ANalysis of COMposition of Microbiomes (ANCOM) (43).

## Results

### Physicochemical Characterisation of Whey Products

**Figure 2:**
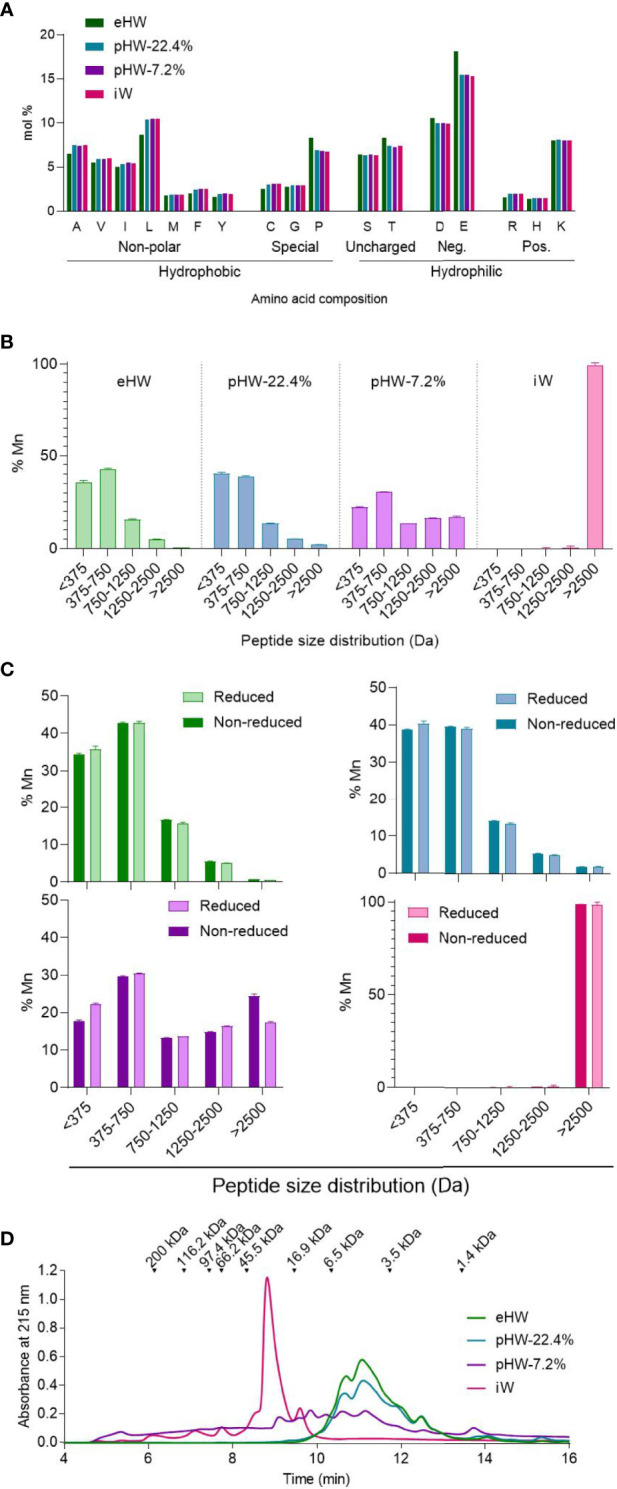
Physicochemical characteristics of whey products. Amino acid distribution **(A)**, peptide size distribution under reduced condition (disulphide bonds and non-covalent interactions disassociated) alone **(B)** or in comparison to the non-reduced condition (non-covalent interactions disassociated) **(C)**, and aggregation status under physiological conditions (no interactions disassociated) **(D)** of extensively (eHW), partially hydrolysed (pHW-22.4% or pHW-7.2%) or intact (iW) whey products. Mean and standard deviation of triplicates **(B, C)**. Mn, number average molecular mass.

Four protein ingredients for IF were included in the study: One intact whey product (iW), two partially hydrolysed whey products (pHW-7.2% and pHW-22.4%) and one extensively hydrolysed whey product (eHW) with degree of hydrolysis of 7.2%, 22.4% and 27%, respectively. Only eHW had been filtrated after hydrolysis and thus had a slightly different AA composition compared to pHW-22.4%, pHW-7.2% and iW, for which the AA composition was similar (**Figure 2A**).

The products iW and pHW-7.2% both had a unique peptide size distribution profile (**Figure 2B**). The two products with the highest degree of hydrolysis, eHW and pHW-22.4%, had similar profiles except that peptides above 2500 Da (approx. 22 AAs) were absent in eHW (**Figure 2B**).

For pHW-7.2%, notable differences were observed between peptide size distributions analysed under reduced and non-reduced conditions (**Figure 2C**). This indicates that complexes are formed by disulphide bonds between multiple small peptides, especially those below 375 Da (approx. 4 AAs) in this product. The presence of protein complexes in pHW-7.2% was confirmed by gel permeation chromatography under physiological conditions (**Figure 2D**). Under physiological conditions, small complexes with sizes below 5.5 kDa were also observed in eHW and pWH 22.4% (**Figure 2D**).

### Partially Hydrolysed Whey Products Prevent Whey Sensitisation and Allergic Reactions

The four different whey products were administered to naïve BN rats (with a conventional microbiota) in their drinking water for three weeks. Rats were subsequently ip post-immunised with iW once a week for four weeks to evaluate the capacity of the products to reduce whey-specific sensitisation and allergic reactions to intact whey (**Figure 1**).

**Figure 3:**
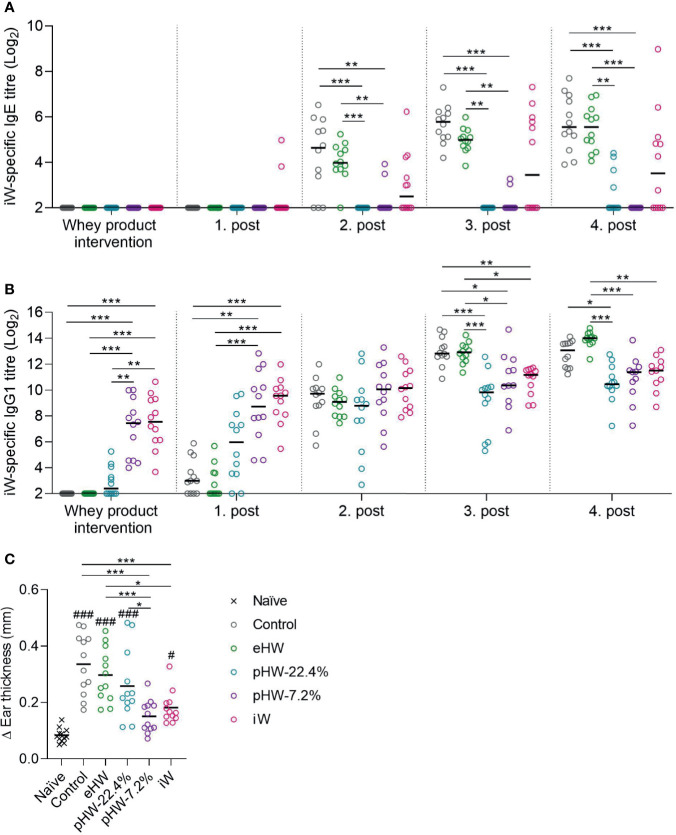
Preventive capacity of the whey protein products. Intact whey (iW)-specific IgE titres **(A)**, iW-specific IgG1 titres **(B)**, and Δear swelling after ear-swelling test **(C)** in rats that received extensively (eHW), partially hydrolysed (pHW-22.4% or pHW-7.2%) or intact (iW) whey products in drinking water or water alone as control. Analyses were performed on sera after whey product interventions on Day 35 and after the post immunisations on Day 42, 49, 56 and 64. Each symbol represents a single rat and horizontal lines indicate median **(A, B)** or mean **(C)** values. Statistically significant differences between product groups are indicated as *p ≤ 0.05, **p ≤ 0.01, ***p ≤ 0.001, and between product groups and naïve controls as ^#^p ≤ 0.05 and ^###^p ≤ 0.001.

Control rats receiving no product in their drinking water were readily sensitised to whey after two to three ip post-immunisations (**Figure 3A**). In the eHW group, a transient reduction in whey-specific IgE level compared to the control group was observed after three post-immunisations (p=0.025, t-test between eHW and control groups). In both the pHW-22.4% and pHW-7.2% groups strong prevention was observed since the whey-specific IgE level in those groups remained significantly lower compared to the control group after each post-immunisation. No significant difference in whey-specific IgE level between the pHW-22.4% and pHW-7.2% groups was observed. In the iW group, two out of twelve rats had detectable levels of whey-specific IgE already after the first post-immunisation, indicating that oral exposure to iW had already primed an IgE immune response. Interestingly, from the second post-immunisation, the whey-specific IgE response in the iW group was very heterogeneous and consequently not significantly different from the control group. Here, six to seven out of twelve rats had detectable levels of whey-specific IgE, which was shown to be significant higher than the IgE levels in the pHW-22.4% group (p=0.014 after post-immunisation 2, p=0.015 after post-immunisation 3, non-parametric Mann-Whitney test) and pHW-7.2% group (p=0.033 after post-immunisation 3, p=0.005 after post-immunisation 4, non-parametric Mann-Whitney test).

Analysis of whey-specific IgG1, as a measure of the general immune response mounted upon antigen dosing, levels after intervention with whey products and first post-immunisation revealed that the immunogenicity varied between the products; the highest IgG1 level was observed in the iW and pHW-7.2% groups, a lower level in the pHW-22.4% group and lowest in the eHW group (**Figure 3B**). This finding is in line with the notion that hydrolysis eliminates the antigenic epitopes of whey.

One week after the fourth post-immunisation (Day 63), allergic reactions were assessed by an ear-swelling test by intradermal injection of iW. The iW and pHW-7.2% groups had the lowest ear swelling, and these two groups were the only ones significantly lower than the control group (**Figure 3C**). The ear swelling in the pHW-7.2% group was, contrary to the iW group, not even significantly different from the non-specific swelling observed in naïve rats subjected to the same ear-swelling test.

The ear swelling results (**Figure 3C**) generally reflect the IgE results (**Figure 3A**). Accordingly, the IgE levels correlated with the ear swelling response in the eHW and pHW groups (Spearman’s correlation, p < 0.001). Within the iW group some of the rats showed only little ear-swelling despite having high IgE levels. This could indicate that the raised IgE antibodies had decreased functionality or that IgE function was blocked by other antibody classes.

### Amoxicillin Reduces Microbial Diversity and Eradicates Commensal Species

To investigate whether antibiotic-associated perturbation of the gut microbiota composition affects allergy prevention, parallel groups of rats were administered with amoxicillin by daily oral gavage from one week before and during the intervention with whey products (**Figure 1**). The effect of amoxicillin on microbiota composition was analysed by comparing all amoxicillin administered groups with all conventional groups (water administered).

**Figure 4:**
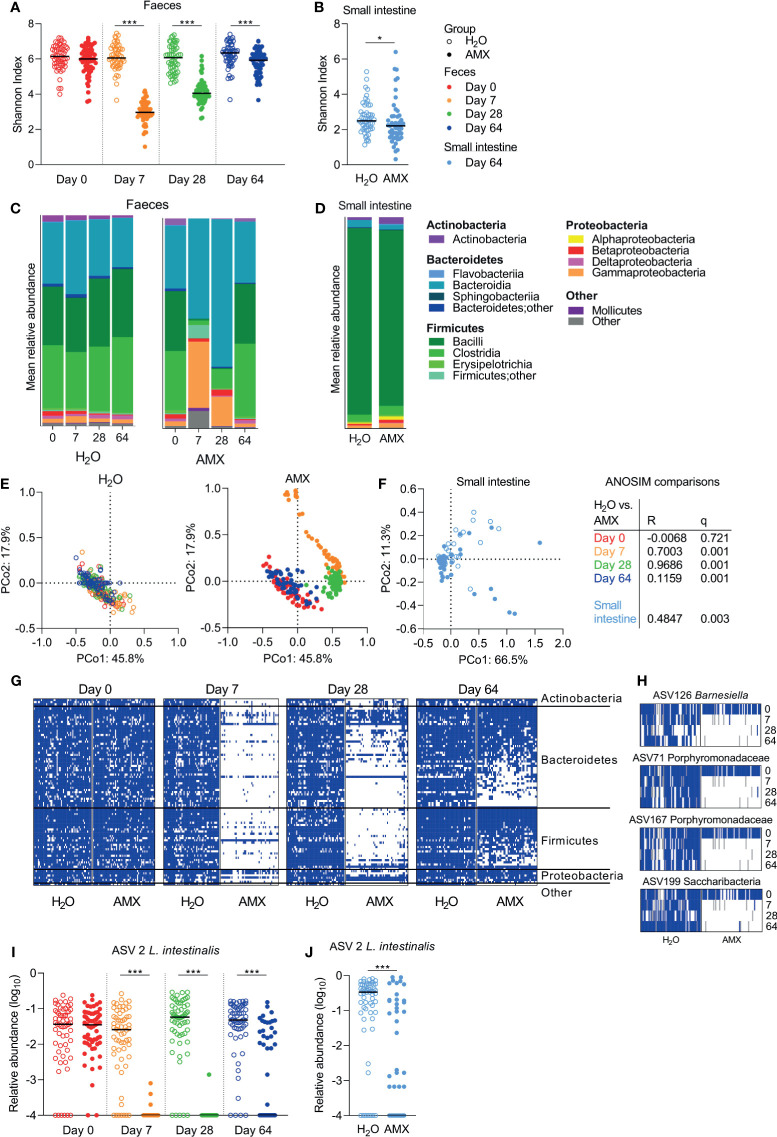
Effects of amoxicillin on faecal and small intestine microbiota. Shannon diversity index of faecal **(A)** and small intestinal **(B)** microbiota of conventional (H_2_O, open symbols) and amoxicillin (AMX, closed symbols) groups. Mean relative abundance of bacterial classes in faecal **(C)** and small intestinal **(D)** microbiota. Principal coordinate analysis (PCoA) plots of weighted UniFrac distances in faecal **(E)** and small intestinal **(F)** samples are coloured according to sample day. Pairwise comparisons between the H_2_O and AMX groups at different days in faeces and in small intestine by analysis of similarities (ANOSIM). Presence/absence status of the most prevalent amplicon sequencing variant (ASVs), present in ≥75% of the rats on Day 0, in the faecal samples at different days **(G)**. Presence (blue)/absence (white)/missing data (grey) status of four ASVs at different days **(H)**. Relative abundance of *Lactobacillus intestinalis* in faecal **(I)** and small intestinal **(J)** microbiota. Each symbol represents a single rat and horizontal lines indicate median **(A, B, H, I)** values. Statistically significant differences between groups are indicated as *p ≤ 0.05, ***p ≤ 0.001.

Within sample (α) diversity, as determined by the Shannon diversity index, was significantly lower in faeces (**Figure 4A**) and the small intestine (**Figure 4B**) in the amoxicillin groups compared to the conventional groups. In faecal samples, the effect was most pronounced on Day 7, and gradually decreased hereafter, but remained significant throughout the experiment, which ended five weeks after cessation of amoxicillin administration (Day 64) (**Figure 4A**).

After acute (Day 7) and prolonged (Day 28) exposure to amoxicillin, the faecal mean relative abundances of Firmicutes and Actinobacteria were lower in the amoxicillin groups than in the conventional groups, while the relative abundances of Proteobacteria, especially Gammaproteobacteria, and Bacteroidetes were higher (**Figure 4C**). Five weeks after cessation of amoxicillin administration (Day 64), the overall microbial composition in faeces (**Figure 4C**) and small intestinal content (**Figure 4D**) was similar to that of the conventional groups. The small intestine was vastly dominated by *Lactobacillus* (Bacilli), and this genus constituted on average 84.1% and 76.4% of bacteria in the water and amoxicillin groups, respectively, however with a great variation between individual rats (**Figure S2**).

In line with the observed longitudinal effects of amoxicillin on bacterial phylum distribution, the between sample (β) diversity assessed by ASV-based principal coordinate analysis (PCoA) of unweighted UniFrac distances revealed a temporal shift in faecal microbial composition of the amoxicillin groups during the study (**Figure 4E**). On Day 64, the microbial composition of amoxicillin rats approached that of Day 0, but did not revert completely, and remained significantly different from the conventional groups as determined by ANOSIM in the faecal (**Figure 4E**) and the small intestinal (**Figure 4F**) microbiota. This persistent difference between the amoxicillin and conventional groups was mostly due to some ASVs that were absent after cessation of antibiotic treatment (**Figure 4G**). Out of the most prevalent ASVs (present in ≥75% of all samples on Day 0), four ASVs were almost eradicated and present in <2% of samples in the amoxicillin groups (**Figure 4H**). Three of these were assigned to the family Porphyromonadaceae (Bacteroidetes) of which one was further assigned to the genus *Barnesiella.* One ASV was assigned to the phylum *Candidatus* Saccharibacteria (“Other”). Numerous ASVs were differently abundant in the amoxicillin and conventional groups in the faecal microbiota (**Table S3**), while only two ASVs were differently abundant in the small intestinal microbiota (**Table S4**) as determined by ANCOM. Of these, one ASV assigned to the species *Lactobacillus intestinalis*, varied significantly in abundance between the amoxicillin and conventional groups in both faeces and small intestines (**Figures 4I, J**).

### Amoxicillin Promotes Helios^-^ Regulatory T Cells and Acute Faecal IgA Responses

Host intestinal immune regulation was assessed by quantifying IgA in faeces and sera throughout the experiment and by profiling various lymphocyte populations at experiment termination. The effect of amoxicillin on host intestinal immune regulation was assessed by comparison of all amoxicillin groups with all conventional groups.

**Figure 5:**
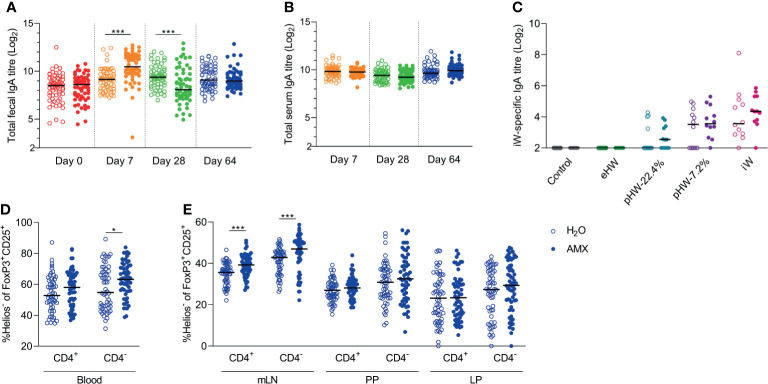
Effect of amoxicillin administration on host immune regulation. Total faecal **(A)** and total serum **(B)** IgA titres in conventional (H_2_O, open symbols) or amoxicillin (AMX, closed symbols) groups. Intact whey (iW)-specific serum IgA titres on Day 28 in rats administered with extensively (eHW), partially hydrolysed (pHW-22.4% or pHW-7.2%) or intact (iW) whey products or water as control in their drinking water **(C)**. Fraction of Helios^-^ Tregs out of the CD25^+^FoxP3^+^ Treg populations (both CD4^+^ and CD4^-^) in blood **(D)**, and mesenteric lymph nodes (mLN), Peyer’s patches (PP) and lamina propria (LP) on Day 64 **(E)**. Each symbol represents a single rat and horizontal lines indicate median values. Statistically significant differences between groups are indicated as *p ≤ 0.05, ***p ≤ 0.001.

The level of total faecal IgA was affected by amoxicillin administration in a time-dependent manner; on Day 7, the level was significantly higher in the amoxicillin groups than in the conventional groups, whereas on Day 28, it was significantly lower (**Figure 5A**), indicating that acute changes in microbiota composition or amoxicillin directly regulate local IgA secretion. Five weeks after amoxicillin cessation (Day 64), no difference in total faecal IgA levels was observed between the amoxicillin groups and conventional groups. Total serum IgA level was not affected by amoxicillin administration (**Figure 5B**) and no statistically significant difference in levels of whey-specific serum IgA in amoxicillin administered rats compared to conventional rats was observed after the intervention with whey products (**Figure 5C**). We have previously observed an acute induction of faecal IgA and LP regulatory T cells (Tregs) after 7 days of amoxicillin administration (34). In the current study, we found no difference in Treg frequencies, nor in the distribution of helper T cells, cytotoxic T cells, or B cells in blood, mLN and small intestinal PP and LP five weeks after cessation of amoxicillin administration (Day 64) (**Figure S3**), suggesting a return to homeostatic levels at this time point. However, the fraction of Helios^-^ Tregs within the CD25^+^FoxP3^+^ Treg population was significantly higher in blood (**Figure 5D**) and mLN (**Figure 5E**) in the amoxicillin groups than in the conventional groups (Day 64), indicating a long-lasting effect of amoxicillin, or amoxicillin-induced changes in the microbiota, on the phenotype of systemic Tregs. Normalising IgA and lymphocyte counts to the median of the water groups of each product group, to account for the small variation between the product groups, did not impact the interpretation of the results (**Figure S4**).

### Amoxicillin Promotes the Prevention of Allergic Reactions in the Extensively Hydrolysed Whey Group

**Figure 6:**
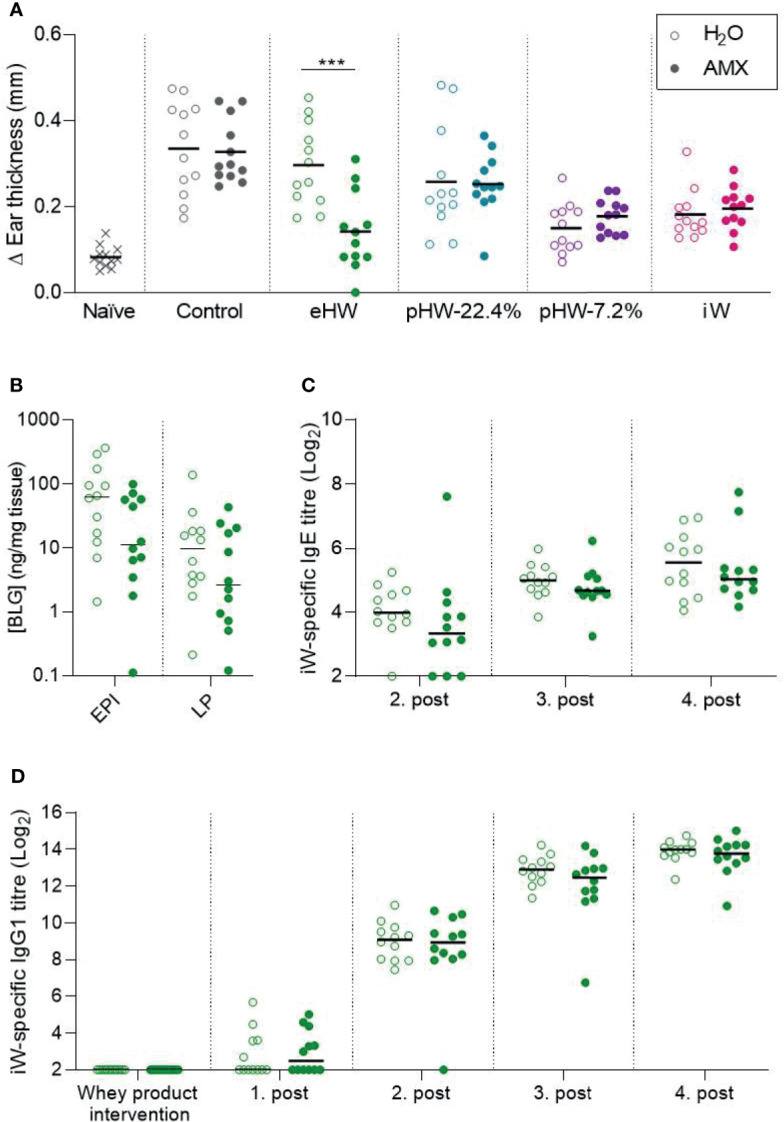
Effect of amoxicillin on the preventive capacity of the whey products. Ear swelling after intradermal injection of intact whey product on Day 63 in water (H_2_O, open symbols) and amoxicillin (AMX, closed symbols) in rats administered with extensively (eHW), partially hydrolysed (pHW-22.4% and pHW-7.2%) or intact (iW) whey products or water as control **(A)**. β-lactoglobulin (BLG) concentration in small intestine epithelium (EPI) and lamina propria (LP) after oral challenge with iW protein **(B)**, iW-specific IgE titres **(C)**, and iW-specific IgG1 titres **(D)**. Subfigures **(B–D)** only show results for the eHW group. Each symbol represents a single rat and horizontal lines indicate mean **(A)** or median **(B–D)** values. Statistically significant differences are indicated as ***p ≤ 0.001.

Despite the fact that amoxicillin administration disturbed the gut microbiota and affected immune regulation, no significant differences in whey-specific sensitisation were observed between amoxicillin and conventional groups within any of the whey product intervention groups, nor in the control groups receiving water (**Figure S5**). However, in the eHW group, ear swelling after injection of intact whey was reduced in the amoxicillin administered rats compared to conventional rats (**Figure 6A**). Surprisingly, this may indicate that amoxicillin administration promoted the prevention of allergic reactions in that group. In line with this, a tendency (p=0.079) for reduced whey protein uptake in the small intestine epithelium was observed (**Figure 6B**), which may indicate reduced allergic reactions. Despite the observed differences in allergic reactions, the whey-specific IgE (**Figure 6C**) or IgG1 (**Figure 6D**) levels were not significantly different between the amoxicillin and conventional groups. No differences in ear-swelling between amoxicillin and conventional rats were observed for any of the other product groups (**Figure 6A**).

### Immunological and Morphological Effects of Amoxicillin in the Extensively Hydrolysed Whey Group

**Figure 7:**
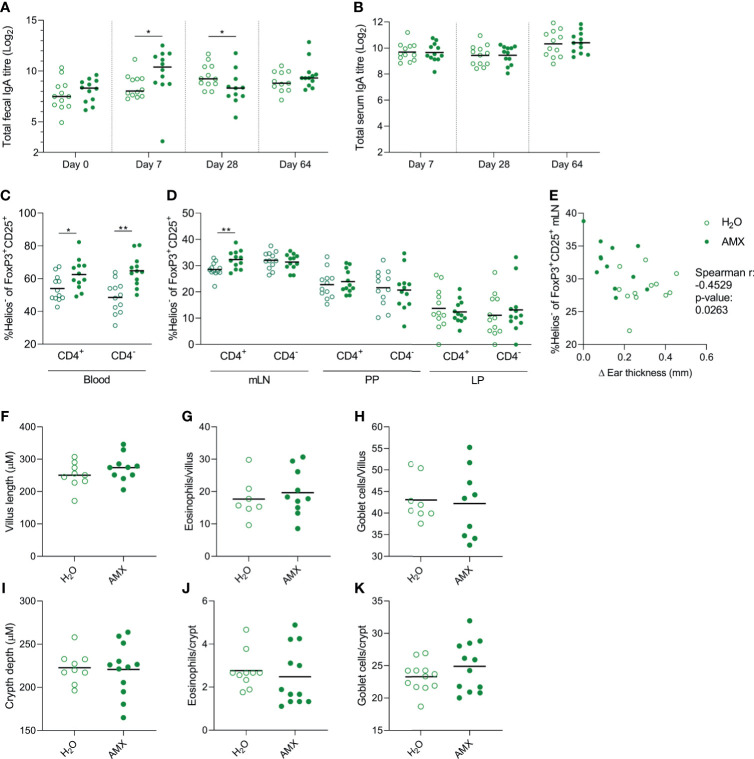
Effect of amoxicillin administration on immune regulation in the extensively hydrolysed whey (eHW) group. Total faecal **(A)** and total serum **(B)** IgA titres in conventional (H_2_O, open symbols) and amoxicillin (AMX, closed symbols) groups. Fraction of Helios^-^ Tregs out of the CD25^+^FoxP3^+^ Treg populations (both CD4^+^ and CD4^-^) in blood **(C)**, and mesenteric lymph nodes (mLN), Peyer’s patches (PP) and lamina propria (LP) **(D)**. Correlation between fraction of Helios^-^ Tregs out of the CD4^+^ Treg populations and ear swelling after intradermal injection of intact whey **(E)**. Average villus length **(F)**, eosinophil **(G)** and goblet cell **(H)** count per villus in small intestine, and average crypt depth **(I)**, eosinophil **(J)** and goblet cell **(K)** count per crypt in colon. Each symbol represents one rat and horizontal lines indicate median **(A)** or mean **(B–K)** values. Statistically significant differences between groups are indicated as *p ≤ 0.05, **p ≤ 0.01.

As amoxicillin seemed to promote prevention of allergic symptoms in the eHW group, the effect of amoxicillin administration on host immune regulation was assessed by reanalysing data for immune regulation in this group separately. The result for total faecal IgA level (**Figure 7A**), total serum IgA level (**Figure 7B**) and Helios^-^ Tregs frequency (**Figures 7C, D**) in the eHW group alone confirmed the summed observations across all whey product groups (**Figure 5**). Furthermore, a significant inverse correlation between allergic reactions measured by the ear-swelling test and Helios^-^ CD4^+^Tregs frequency in mLN (**Figure 7E**) was observed, indicating that the preventive effect of amoxicillin may involve activation of Helios^-^ Tregs.

Additionally, possible morphological effects of amoxicillin were assessed by histological analysis. No effects of amoxicillin on small (**Figure 7F**) or large intestinal (**Figure 7I**) tissue morphology, eosinophil (**Figures 7G, J**) or goblet cell (**Figures 7H, K**) count was observed five weeks after cessation of amoxicillin administration (Day 64).

### Partially Hydrolysed Whey Products Promote *In Vitro* Expansion of *Enterococcus*

To investigate whether the different products affected gut microbiota composition directly, faecal samples from three human healthy infant donors and one defined mixed culture prepared from equal amounts of three common human infant gut bacteria (*B. longum* ssp. *Infantis, L. rhamnosus, E. faecalis)*, were anaerobically incubated in minimal medium supplemented with the four different whey products separately.

**Figure 8:**
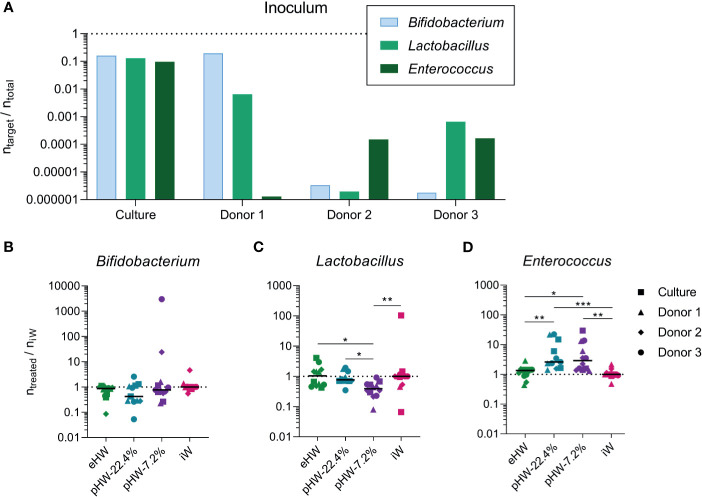
Effect of whey products on *in vitro* expansion of bacterial genera derived from healthy infants. The relative abundance of selected bacterial genera before **(A)** and after **(B–D)** *in vitro* incubations supplemented with extensively (eHW), partially hydrolysed (pHW-22.4% or pHW-7.2%) or intact (iW) whey products. The results after *in vitro* incubations **(B–D)** are normalised to the median of the iW group. Each bar represents one donor/culture **(A)**, each symbol represents an *in vitro* incubation replicate, which is the average of three technical qPCR replicates and horizontal lines indicate median values **(B–D)**. The symbol shape indicates the inoculum source. Statistically significant differences between groups are indicated as *p ≤ 0.05, **p ≤ 0.01, ***p ≤ 0.001.

The three healthy, exclusively breastfed human infant donors each had different relative abundances of the three analysed bacterial genera (**Figure 8A**). The response was very variable across the different donors for *Bifidobacterium* and *Lactobacillus*, while for *Enterococcus* the response was uniform across the three donors (**Figure S6**). Across the donors and defined mixed culture, no significant effect on the relative abundance of *Bifidobacterium* was observed of any product (**Figure 8B**). The relative abundance of *Lactobacillus* was lower in incubations supplemented with pHW-7.2% compared to all other products (**Figure 8C**). The relative abundance of *Enterococcus* was higher in incubations supplemented with pHW-22.4% and pHW-7.2% compared to both eHW and iW (**Figure 8D**). For *Enterococcus*, no difference between the products was observed for the defined culture (**Figure S6F**), hence it is likely not the growth of *E. faecalis* specifically that was affected by the partially hydrolysed products.

## Discussion

### Partially Hydrolysed Whey Products Prevent Whey Sensitisation and Allergic Reactions

The use of hydrolysed IFs for allergy prevention has been suggested as a good option for atopy-prone infants due to their reduced allergenicity, but is still highly debated due to inconsistent results from clinical trials (7). We used a previously established animal model (20, 36) to compare whey-based IFs with different physiochemical properties. Our study revealed that the non-filtered partially hydrolysed products, pHW-22.4% and pHW-7.2%, with very different peptide size distribution and aggregation profiles, were both very efficient in preventing subsequent whey-specific sensitisation and allergic reactions to iW. In contrast, the filtered, extensively hydrolysed product eHW, with degree of hydrolysis on 27% and a peptide size distribution that only marginally differed from that of pHW-22.4%, had poor preventive capacity. Small-size complexes were observed in both eHW and pHW-22.4% under physiological conditions, but since these complexes were of identical sizes in the two products, these cannot explain the difference in the preventive capacity between the eHW and pHW-22.4%. The reduced preventive capacity of eHW compared to the other hydrolysed products may likely be related to the removal of larger peptides above 2.5 kDa from eHW by filtration.

In the eHW group, a statistically significant reduction in IgE level compared to the control group was observed only transiently, and no difference in IgE level nor ear-swelling was observed at the end of the study. Similarly, previous animal studies have shown that eHF retains only marginal capacity to prevent sensitisation and allergic reactions, and much less than pHF (17, 19, 20). In line with our results, two previous reports indicate that administration of partially hydrolysed whey products can completely prevent epicutaneous (19) and ip (20) sensitisation. Other studies found that partially hydrolysed whey products reduced oral (16, 18) or ip (17) sensitisation but not as efficiently as intact products. However, in most of these studies the partially hydrolysed whey products used were not well characterised.

In the current study, the level of whey-specific IgE was lower in rats that received partially hydrolysed products prior to sensitisation compared to iW. A very heterogeneous whey-specific IgE response was observed in the iW group, where some rats had detectable levels of whey-specific IgE already after the first post-immunisation. This indicates that oral exposure to iW had primed an IgE response. Other rats in the same group were well protected and never raised detectable levels of whey-specific IgE even after four post-immunisations. The whey-specific IgE level was not significantly different between the iW group and the control group, which did not receive any product for prevention. To our knowledge, the current study is the first to show that hydrolysed whey is superior to intact whey in preventing whey-specific sensitisation. These results support the use of partially hydrolysed whey, with a reduced sensitising capacity, to infants at high risk of developing CMA. However, these results warrant further investigation to confirm translation to infants.

### Amoxicillin Reduces Microbial Diversity and Eradicates Commensal Species

Amoxicillin induced a major shift in the faecal microbiota, characterised by reduction in α diversity, reduction in relative abundance of Firmicutes and Actinobacteria, and increased relative abundance of Gammaproteobacteria and Bacteroidetes. These effects are in accordance with our previous observation of the effect of seven-days amoxicillin administration in BN rats (34), as well as with the reported effect in healthy human adults (44, 45).

Five weeks after cessation of amoxicillin administration, the effect of amoxicillin on both α and β diversities were subtle, but significant differences remained. Observations from human studies showed that faecal microbiome perturbations after antibiotic administration recovered to near baseline within 1-2 weeks, but that differences in certain taxa persisted for six months to two years after antibiotic cessation (46–49). Only few studies have investigated the long-term consequences of intervention with amoxicillin. A recent large study in healthy human adults reported that gut microbiota did recover to baseline in terms of β diversity already one week after amoxicillin cessation, but noted that three operational taxonomic units were still different from the baseline relative abundance two weeks after cessation (45). The more persistent effects of amoxicillin treatment observed in the current study on overall composition and individual taxa may in part be explained by the animals’ young age. In support of this, it is known that the time it takes for children’s microbiota to return to baseline after antibiotic treatment is longer than for adults (50).

### The Effect of Amoxicillin on Allergy Prevention Likely Involves Bacteria-Induced iTregs and IgA

Even though daily amoxicillin administration significantly disturbed the gut microbiota, this did not impair prevention of sensitisation or allergic reaction. No significant difference in prevention of whey-specific sensitisation was observed between amoxicillin and conventional groups. Ear-swelling results even suggested that amoxicillin administered rats were better protected against allergic reactions than those with a conventional microbiota within the eHW groups, despite similar whey-specific IgE levels. In light of previous studies in mice indicating that microbiota depletion with broad-spectrum antibiotic cocktails promotes sensitisation (51–53), this was unexpected. However, most sensitisation models require non-physiological methods of sensitisation to allergens such as damaging the gastrointestinal tract with cholera toxin, for which the mucosal adjuvant function has been shown to be microbiota dependent (54). A strength of our study is that the effect of microbiota perturbation on allergy prevention was investigated under physiologically relevant conditions by administering whey products in the drinking water similarly to feeding of IF without the use of any adjuvant. All rats were subsequently subjected to non-physiologically relevant ip immunisations. Accordingly, sensitisation was not affected by the microbiota perturbation, and the effect of the amoxicillin intervention on the prevention phase could be pinpointed.

Only two studies have previously assessed the influence of antibiotic-induced microbiota perturbation on allergy prevention (55, 56), and in accordance with our results, these found that administration of amoxicillin before and during interventions with ovalbumin either slightly promoted or did not affect prevention of ovalbumin allergy (55).

It is well recognised that Tregs play an important role in tolerance development (57). Tregs are either produced in the thymus (natural, nTreg) or induced in the peripheral tissues (induced, iTreg). The iTreg population can be distinguished from nTregs by their low expression of the transcription factor Helios (58). Recent studies suggest that commensal bacteria-induced Helios^-^ROR-γt^+^ iTregs play an important role in oral tolerance development and in the prevention of food allergy in human and murine models (27). It has also been found that Helios^-^ROR-γt^+^ iTregs were dramatically reduced in mice treated with a broad-spectrum antibiotic cocktail (59, 60), but were less reduced or unaffected by treatment with each individual antibiotic (59), implying some degree of redundancy in microbial activation of Helios^-^ROR-γt^+^ iTregs.

Results from the current study indicate that the microbiota perturbation induced by administration of a single clinically relevant antibiotic, namely amoxicillin, actually increased the frequency of Helios^-^ iTregs in mLNs and blood. This was associated with acute increased total faecal IgA, and is in line with our previous observation of acute upregulation of a Treg-IgA axis after amoxicillin administration (34). The tolerance promoting effect of amoxicillin in the eHW group is likely related to the upregulation of the iTreg-IgA axis locally in the gut, but other factors not investigated in the study, such as direct immunomodulatory effects or effects of microbiota-perturbation on digestion and intestinal uptake of proteins during whey protein interventions, may potentially also have contributed to the result. Our previous results however suggests that amoxicillin does not significantly affect intestinal uptake of cow’s milk proteins in naïve rats (34).

### Partially Hydrolysed Whey Products Promote *In Vitro* Expansion of *Enterococcus*

Even though products based on hydrolysed whey are commonly used for infants suffering from CMA or at high risk of developing CMA, only few studies have addressed the effect of hydrolysed cow’s milk protein-based IF on gut microbiota (61, 62). To address this, an *in vitro* incubation study was conducted with faecal samples from three healthy human infant donors and one defined culture of three common infant gut bacteria, which were anaerobically incubated in minimal medium supplemented with each of the whey products.

While the three infant donors’ initial microbiotas were very different, they responded quite uniformly to *in vitro* incubation with regard to the relative abundance of *Enterococcus*. The relative abundance of *Enterococcus* was higher in the incubations with pHW-22.4% and pHW-7.2% compared to eHW and iW. *Enterococcus* species are generally considered to have a very efficient proteolytic system (63), and the species present in the samples were thus more efficient in utilising the partially hydrolysed whey compared to intact whey. The response was similar for all three donors, while no response was observed for the defined culture, probably because the specific strain of *E. faecalis* in the synthetic culture mix was different from the enterococci present in the donors’ microbiotas.

Species belonging to *Enterococcus* are common gut bacteria in early life, and have been detected in both meconium of healthy infants and in human breast milk (64, 65). However, children sensitised to food allergens have been reported to have higher relative abundances of *Enterococcus* than healthy children (66), and high relative abundance of *Enterococcus* in early life has been associated with respiratory problems in infants (67). The impact of whey products on this bacterial group should thus be considered.

In summary, oral administration of two partially hydrolysed whey products with different physicochemical characteristics were found to be superior in preventing whey-specific sensitisation compared to extensively hydrolysed and intact whey products, though intact whey was as efficient as the partially hydrolysed whey products in preventing clinical reactions. Amoxicillin administration disturbed the gut microbiota but did not impair the preventive effect of the whey products. The *in vitro* incubation of infant faecal samples with the whey products indicated that partially hydrolysed whey products might confer a selective advantage to enterococci growth. Collectively, our results support the use of partially hydrolysed whey-based IF for CMA prevention in infants predisposed to develop CMA regardless of their host microbiota status. However, the direct effect of hydrolysed products on infant microbiota composition, revealed by the *in vitro* incubation study with infant-derived faecal samples, warrants further investigation.

## Data Availability Statement

The datasets presented in this study can be found in online repositories. The names of the repository/repositories and accession number(s) can be found below: https://www.ncbi.nlm.nih.gov/, PRJNA691570.

## Ethics Statement

Faecal samples were obtained from three healthy human infant donors. All donors were 1-4 months old, exclusively breastfed, and had never received antibiotics. The study was approved by The Danish National Committee on Health Research Ethics (H-16030078), and written informed consent was obtained from the parents of the faecal donors. Ethical approval for the animal experiment was given by the Danish Animal Experiments Inspectorate (2015-15-0201-00553-C1). The experiments were overseen by the National Food Institute’s in-house Animal Welfare Committee for animal care and use.

## Author Contributions

KB and TL conceived the study. KG, MB, TL, and KB designed the animal study and participated in the interpretation of all results. LS, HC, and LJ selected the whey products and characterised them, which included the development of new experimental methods. KG and SP performed the animal experiment (in collaboration with animal care takers and lab technicians), analysed serum antibody levels, histology and protein uptake. KG and SP analysed microbiota data in close collaboration with MB. JL analysed lymphocyte populations and participated in interpretation of the results. KG, MB, TL, and KB designed the *in vitro* study. SH participated in formulating participant inclusion criteria, information materials etc. KG performed the *in vitro* incubation study and analysed the results in close collaboration with MB. KG drafted the manuscript. All authors contributed to the article and approved the submitted version.

## Funding

This work was supported by the Danish Dairy Research Foundation.

## Conflict of Interest

LJ, HC, and LS are employees at Arla Foods Ingredients. KB has ongoing collaboration with the company Arla Foods Ingredients, which supplied the whey protein products for the current study.

The remaining authors declare that the research was conducted in the absence of any commercial or financial relationships that could be construed as a potential conflict of interest.

## Publisher’s Note

All claims expressed in this article are solely those of the authors and do not necessarily represent those of their affiliated organizations, or those of the publisher, the editors and the reviewers. Any product that may be evaluated in this article, or claim that may be made by its manufacturer, is not guaranteed or endorsed by the publisher.
