# Supplementary material for: Partially Hydrolysed Whey Has Superior Allergy Preventive Capacity Compared to Intact Whey Regardless of Amoxicillin Administration in Brown Norway Rats
Source: Front Immunol. 2021 Aug 31;12:705543. doi: 10.3389/fimmu.2021.705543 (PMC8438296; doi:10.3389/fimmu.2021.705543)
Supplement: Supplementary file 1 [file DataSheet_1.pdf]

Supplementary Material

1 Supplementary Methods

1.1 Product characterisation

1.1.1 Degree of hydrolysis

The degree of hydrolysis was determined with an *O*-Phthaldialdehyd (OPA)-assay as described elsewhere (1). Briefly, samples were dissolved to a concentration of 0.8 mg/mL protein in milli Q water and filtered through filter paper, and subsequently 20 µL sample was mixed with 150 µL OPA reagent (0.08% O-Phthaldialdehyd (Merck KGaG, Darmstadt, Germany), 2% (v/v) ethanol (VWR International, Radnor, PA, US), 3.81% di-sodium-tetraborat-decahydrat (Merck KGaG), 0.1% Sodium-dodecyl-sulfat (VWR International), 0.088% DL-Dithiothreitol (Merck KGaG)). Absorbance was measured after 120 s at 340 nm on a Lambda 35 UV/VIS Spectrometer (Perkin Elmer, Waltham, MA, US). A calibration curve based on L-serine (Merck KGaG) diluted in milli Q water was prepared in the range of 0.067-0.28 mg/mL.

1.1.2 Amino acid composition

AA analysis was performed by ion exchange chromatography after hydrolysis in HCl, as described elsewhere (2).

1.1.3 Peptide size distribution

Peptide size distribution was determined by TSK gel® permeation chromatography (GPC). Samples were dissolved to a concentration of 0.32% (w/v) protein in mobile phase buffer (0.0375 M phosphate buffer (VWR International) with 0.375 M NH_4Cl (VWR International), 0.1% trifluoro acetic acid (Merck KGaG), 25% acetonitrile (Rathburn Chemicals, Walkerburn, Scotland)) to disassociate non-covalent interactions. Disulphide bonds were reduced by addition of 0.67% of β-mercaptoethanol (Merck, KGaG) followed by a heat treatment in a heating block at 100 °C for 30 min. All samples were filtrated through a 0.45 µm filter. Chromatographic separation was performed at 25°C on a Thermo Ultimate 3000 LC (Thermo Scientific, Waltham, MA, US) equipped with three TSK G2000 SWXL columns (5 µm 7.8 mm x 300 mm, TOSOH Bioscience GmbH, Stuttgart, Germany) connected in series. Samples were separated at a flow rate of 0.7 mL/min with mobile phase buffer. Peptides/proteins were detected at 214 nm. The relative distribution of peptides was calculated based on area under the curve.

1.1.4 Analysis of protein aggregation

To examine protein aggregation status under physiological conditions (solvent: 50 mM NaH_2PO_4, 0.15 M NaCl, pH 7.0), ethylene bridged hybrid (BEH) GPC was performed as previously described (3).

1. 2 DNA extraction and amplicon sequencing of the 16S rRNA gene

DNA was extracted from faeces or small intestine content by DNeasy PowerLyzer PowerSoil Kit (Qiagen, Hilden, Germany) according to the manufacture's protocol. Mechanical lysis of bacteria was conducted at 30 cycles/s twice for 5 min using bead beater MM300 (Retsch, VWR, Haan, Germany).

The V3-region of the 16S rRNA gene was amplified using a universal forward primer (PBU 5'-A-adapter-TCAG-barcode-CCTACGGGAGGCAGCAG-3') with a unique 10–12 bp barcode for each sample (IonXpress barcode as suggested by the supplier, Life Technologies, Carlsbad, CA, US) and a universal reverse primer (PBR 5'-trP1-adapter-ATTACCGCGGCTGCTGG-3') and Phusion High-Fidelity DNA polymerase (Thermo Fisher Scientific, Waltham, MA, US). PCR products were purified by HighPrep™ PCR Clean-up System (Magbio, Gaithersburg, MD, US) according to the manufacture's protocol. DNA concentrations were determined with Qubit HS assay (Life Technologies). Finally, a library was constructed by mixing an equal amount of PCR products from each sample. Sequencing of all samples was performed on a 318-chip for Ion Torrent sequencing using the Ion OneTouch™ 200 Template Kit v2 DL (Life Technologies).

1.3 Small-scale *in vitro* incubation

1.3.1 Preparation of defined culture mix

Frozen stocks of *Bifidobacterium longum* ssp. infantis (NCIMB 702205), *Lactobacillus rhamnosus* (ATCC 53103) and *Enterococcus faecalis* (DSM 20478) were thawed and plated on Bifidus Selective Medium (BSM) agar plates for 2 days (*B. longum*), de Man, Rogosa and Sharpe (MRS) plates for 2 days (*L. rhamnosus*) or blood agar plates for 1 day (*E. faecalis).* Single colonies of these were inoculated in Gifu Anaerobic Medium (GAM) broth and incubated anaerobically overnight. Finally, the three cultures were mixed to obtain an equal optical density of each and added glycerol in saline to a final concentration around 15% (v/v) and frozen at -80°C in aliquots.

1.3.2 Minimal medium

The minimal basal medium contained: 0.1 g/L of NaCl, 0.04 g/L of K_2HPO_4, 0.04 g/L of KH_2PO_4, 0.01 g/L of $MgSO_4{\cdot}7H2O$, 0.01 g/L of $CaCl_2{\cdot}2H_2O$, 2 g/L of $NaHCO_3$ (all from Merck), 2 g/L of peptone water (Oxoid, Roskilde, Denmark), 1 g/L of yeast extract (Oxoid), 0.5 g/L of bile salts (Oxoid), 2 ml/L of Tween 80 (Sigma) and 0.5 mg/L resazurin (Fluka Honeywell, Charlotte, NC, US) and pH was adjusted to 7.0. After autoclaving, the following sterile filtered ingredients were added: 0.5 g/L of l-cysteine hydrochloride (Sigma), 5 mg/L of hemin (Fluka), 0.001% (v/v) of vitamin K1 (Sigma), and 5 g/L lactose (Merck). The medium was reduced overnight in an anaerobic cabinet.

1.3.3 Real-time PCR conditions

The 16S rRNA-targeting primers used in this study are listed in Table S2. Total reaction volume of 11 µL containing 5.5 µL LightCycler® 480 SYBR Green I Master (Roche), 2.2 pmol of each of the primers (TAG Copenhagen, Denmark), 2 ng template DNA, and nuclease-free water purified for PCR (Qiagen). The reaction conditions were: Pre-incubation at 95°C for 5 min followed by 45 cycles of 95°C for 10 s, 60°C for 15 s and 72°C for 45 s. Lastly, a melting curve was generated (95°C for 5 s, 68°C for 1 min and increasing the temperature to 98°C with a rate of 0.11°C/s with continuous fluorescence detection). The qPCR was run in 384-well format on a LightCycler® 480 II (Roche Applied Science) and analysed using the LightCycler® 480 software.

1.3.4 Real-time PCR data handling

For each incubation replicate, the mean threshold cycle (Ct) value between qPCR triplicates was used to calculate the relative abundance of the genera *Bifidobacterium*, *Lactobacillus* and *Enterococcus* relative to the total bacteria (n_{target}/n_{total}) using $2^{\Delta Ct}$ as described elsewhere (4). ΔCt is the Ct value of the bacterial target normalised to the Ct value of the total bacterial population in the same incubation sample. Furthermore, the ratio between the relative abundance of a bacterial target in incubation with different whey products relative to iW ($n_{treated}/n_{iw}$) were calculated using $2^{\Delta\Delta Ct}$, where ΔΔCt is the ΔCt value of a given sample normalised to the median ΔCt of three iW samples.

2 Supplementary References

1. Nielsen PM, Petersen D, Dambmann C. Improved method for determining food protein degree of hydrolysis. *J Food Sci* (2001) 66:642–646. doi:10.1111/j.1365-2621.2001.tb04614.x

2. Barkholt V, Jensen AL. Amino acid analysis: Determination of cysteine plus half-cystine in proteins after hydrochloric acid hydrolysis with a disulfide compound as additive. *Anal Biochem* (1989) 177:318–322. doi:10.1016/0003-2697(89)90059-6

3. Graversen KB, Ballegaard AR, Kræmer LH, Hornslet SE, Sørensen L V, Christoffersen HF, Jacobsen LN, Untersmayr E, Smit JJ, Bøgh KL. Cow's milk allergy prevention and treatment by heat-treated whey – a study in Brown Norway rats. *Clin Exp Allergy* (2020) 50:708–721. doi:https://doi.org/10.1111/cea.13587

4. Pfaffl MW. A new mathematical model for relative quantification in real-time RT-PCR. *Nucleic Acids Res* (2001) 29:e45.

5. Penders J, Vink C, Driessen C, London N, Thijs C, Stobberingh EE. Quantification of Bifidobacterium spp., Escherichia coli and Clostridium difficile in faecal samples of breast-fed and formula-fed infants by real-time PCR. *FEMS Microbiol Lett* (2005) 243:141–147. doi:10.1016/j.femsle.2004.11.052

6. Heilig HGHJ, Zoetendal EG, Vaughan EE, Marteau P, Akkermans ADL, De Vos WM. Molecular diversity of Lactobacillus spp. and other lactic acid bacteria in the human intestine

as determined by specific amplification of 16S ribosomal DNA. *Appl Environ Microbiol* (2002) 68:114–123. doi:10.1128/AEM.68.1.114-123.2002

7. Walter J, Hertel C, Tannock GW, Lis CM, Munro K, Hammes WP. Detection of Lactobacillus, Pediococcus, Leuconostoc, and Weissella Species in Human Feces by Using Group-Specific PCR Primers and Denaturing Gradient Gel Electrophoresis. *Appl Environ Microbiol* (2001) 67:2578–2585. doi:10.1128/AEM.67.6.2578-2585.2001

8. Rinttilä T, Kassinen A, Malinen E, Krogius L, Palva A. Development of an extensive set of 16S rDNA-targeted primers for quantification of pathogenic and indigenous bacteria in faecal samples by real-time PCR. *J Appl Microbiol* (2004) 97:1166–1177. doi:10.1111/j.1365-2672.2004.02409.x

9. Walter J, Tannock GW, Tilsala-Timisjarvi A, Rodtong S, Loach DM, Munro K, Alatossava T. Detection and identification of gastrointestinal Lactobacillus species by using denaturing gradient gel electrophoresis and species-specific PCR primers. *Appl Environ Microbiol* (2000) 66:297–303. doi:10.1128/AEM.66.1.297-303.2000

3 Supplementary Tables

Table S1. Mean and median ages in days of rats in each experimental group

		Mean	Median
Control	H_2O	29.7	29.5
	AMX	29.7	29.5
eHW	H_2O	35.7	35
	AMX	36.7	38
pHW-22.4%	H_2O	28.2	28
	AMX	28.2	28
pHW-7.2%	H_2O	30.7	31
	AMX	30.4	31
iW	H_2O	28.9	29
	AMX	28.6	29

Table S2. 16S rRNA gene primers used for qPCR in this study

Target	Forward primer (5'-3')	Reverse primer (5'-3')	Amplicon size	Reference
Bifidobacterium	GCGTGCTTAACACATGCAAGTC	CACCCGTTTCCAGGAGCTATT	126 bp	(5)
Lactobacillus	AGCAGTAGGGAATCTTCCA	CACCGCTACACATGGAG	341 bp	(6,7)
Enterococcus	CCCTTATTGTTAGTTGCCATCATT	ACTCGTTGTACTTCCCATTGT	144 bp	(8)
Universal V3	ACTCCTACGGGAGGCAGCAGT	GTATTACCGCGGCTGCTGGCAC	200 bp	(9)

Table S3: Differentially abundant ASVs in faecal samples (Day 64) of amoxicillin administered rats compared to conventional (water administered) rats as determined by ANCOM analysis.

	Taxonomy	W
↓	Bacteria;__Bacteroidetes	1575
	Bacteria;__Firmicutes;__Bacilli;__Lactobacillales;__Lactobacillaceae;__Lactobacillus;__intestinalis	1573
	Bacteria;__Bacteroidetes;__Bacteroidia;__Bacteroidales;__Porphyromonadaceae	1571
	Bacteria;__Bacteroidetes;__Bacteroidia;__Bacteroidales	1568
	Bacteria;__Bacteroidetes;__Bacteroidia;__Bacteroidales;__Porphyromonadaceae	1554
	Bacteria;__Candidatus_Saccharibacteria	1553
	Bacteria;__Bacteroidetes;__Bacteroidia;__Bacteroidales;__Porphyromonadaceae	1546
	Bacteria;__Bacteroidetes;__Bacteroidia;__Bacteroidales;__Porphyromonadaceae	1546
	Bacteria;__Bacteroidetes;__Bacteroidia;__Bacteroidales;__Porphyromonadaceae	1537
	Bacteria;__Bacteroidetes;__Bacteroidia;__Bacteroidales;__Porphyromonadaceae;__Barnesiella	1536
	Bacteria;__Bacteroidetes;__Bacteroidia;__Bacteroidales;__Porphyromonadaceae	1535
	Bacteria;__Firmicutes;__Clostridia;__Clostridiales	1532
	Bacteria;__Bacteroidetes;__Bacteroidia;__Bacteroidales;__Porphyromonadaceae	1520
	Bacteria;__Bacteroidetes;__Bacteroidia;__Bacteroidales;__Porphyromonadaceae	1517
	Bacteria;__Bacteroidetes;__Bacteroidia;__Bacteroidales;__Porphyromonadaceae	1506
	Bacteria;__Bacteroidetes;__Bacteroidia;__Bacteroidales;__Porphyromonadaceae	1504
	Bacteria;__Bacteroidetes;__Bacteroidia;__Bacteroidales;__Porphyromonadaceae;__Barnesiella	1501
	Bacteria;__Bacteroidetes;__Bacteroidia;__Bacteroidales;__Porphyromonadaceae	1499
	Bacteria;__Bacteroidetes;__Bacteroidia;__Bacteroidales	1498
	Bacteria;__Firmicutes;__Bacilli;__Lactobacillales;__Lactobacillaceae;__Lactobacillus;__reuteri	1496
	Bacteria;__Firmicutes;__Bacilli;__Lactobacillales;__Lactobacillaceae;__Lactobacillus	1492
	Bacteria;__Bacteroidetes;__Bacteroidia;__Bacteroidales;__Porphyromonadaceae	1492
	Bacteria;__Bacteroidetes;__Bacteroidia;__Bacteroidales;__Porphyromonadaceae	1489
	Bacteria;__Firmicutes	1484
	Bacteria;__Bacteroidetes;__Bacteroidia;__Bacteroidales;__Porphyromonadaceae;__Barnesiella	1476
	Bacteria;__Bacteroidetes;__Bacteroidia;__Bacteroidales;__Porphyromonadaceae;__Odoribacter	1467
	Bacteria;__Firmicutes;__Clostridia;__Clostridiales;__Ruminococcaceae	1464
	Bacteria;__Bacteroidetes;__Bacteroidia;__Bacteroidales;__Porphyromonadaceae	1460
	Bacteria;__Firmicutes;__Clostridia;__Clostridiales	1456
	Bacteria;__Firmicutes;__Clostridia;__Clostridiales;__Ruminococcaceae	1450
	Bacteria;__Firmicutes;__Clostridia;__Clostridiales;__Lachnospiraceae	1447
	Bacteria;__Firmicutes;__Clostridia;__Clostridiales;__Lachnospiraceae	1441
	Bacteria;__Firmicutes	1436

	Bacteria;__Firmicutes;__Clostridia;__Clostridiales	1432
	Bacteria;__Firmicutes;__Clostridia;__Clostridiales;__Ruminococcaceae	1432
	Bacteria;__Firmicutes;__Clostridia;__Clostridiales;__Lachnospiraceae	1423
↑	Bacteria;__Bacteroidetes;__Bacteroidia;__Bacteroidales;__Rikenellaceae	1580
	Bacteria;__Bacteroidetes;__Bacteroidia;__Bacteroidales	1535
	Bacteria;__Bacteroidetes;__Bacteroidia;__Bacteroidales;__Bacteroidaceae;__Bacteroides	1534
	Bacteria;__Firmicutes;__Clostridia;__Clostridiales	1515
	Bacteria;__Bacteroidetes;__Bacteroidia;__Bacteroidales;__Porphyromonadaceae;__Barnesiella	1488
	Bacteria;__Bacteroidetes;__Bacteroidia;__Bacteroidales;__Bacteroidaceae;__Bacteroides	1480
	Bacteria;__Bacteroidetes;__Bacteroidia;__Bacteroidales;__Bacteroidaceae;__Bacteroides;__acidifaciens	1473
	Bacteria;__Bacteroidetes;__Bacteroidia;__Bacteroidales;__Porphyromonadaceae	1460
	Bacteria;__Firmicutes;__Clostridia;__Clostridiales;__Lachnospiraceae;__Lachnoanaerobaculum	1452
	Bacteria;__Firmicutes;__Clostridia;__Clostridiales;__Ruminococcaceae	1448
	Bacteria;__Bacteroidetes;__Bacteroidia;__Bacteroidales;__Porphyromonadaceae;__Parabacteroides;__goldsteinii	1441

Table S4: Differentially abundant ASVs in small intestine samples of amoxicillin administered rats compared to conventional (water administered) rats as determined by ANCOM analysis

	Taxonomy	W
↓	Bacteria;__Firmicutes;__Bacilli;__Lactobacillales;__Lactobacillaceae;__Lactobacillus;__intestinalis	1022
	Bacteria;__Firmicutes;__Bacilli;__Lactobacillales;__Lactobacillaceae;__Lactobacillus;__reuteri	1021

4 Supplementary Figures

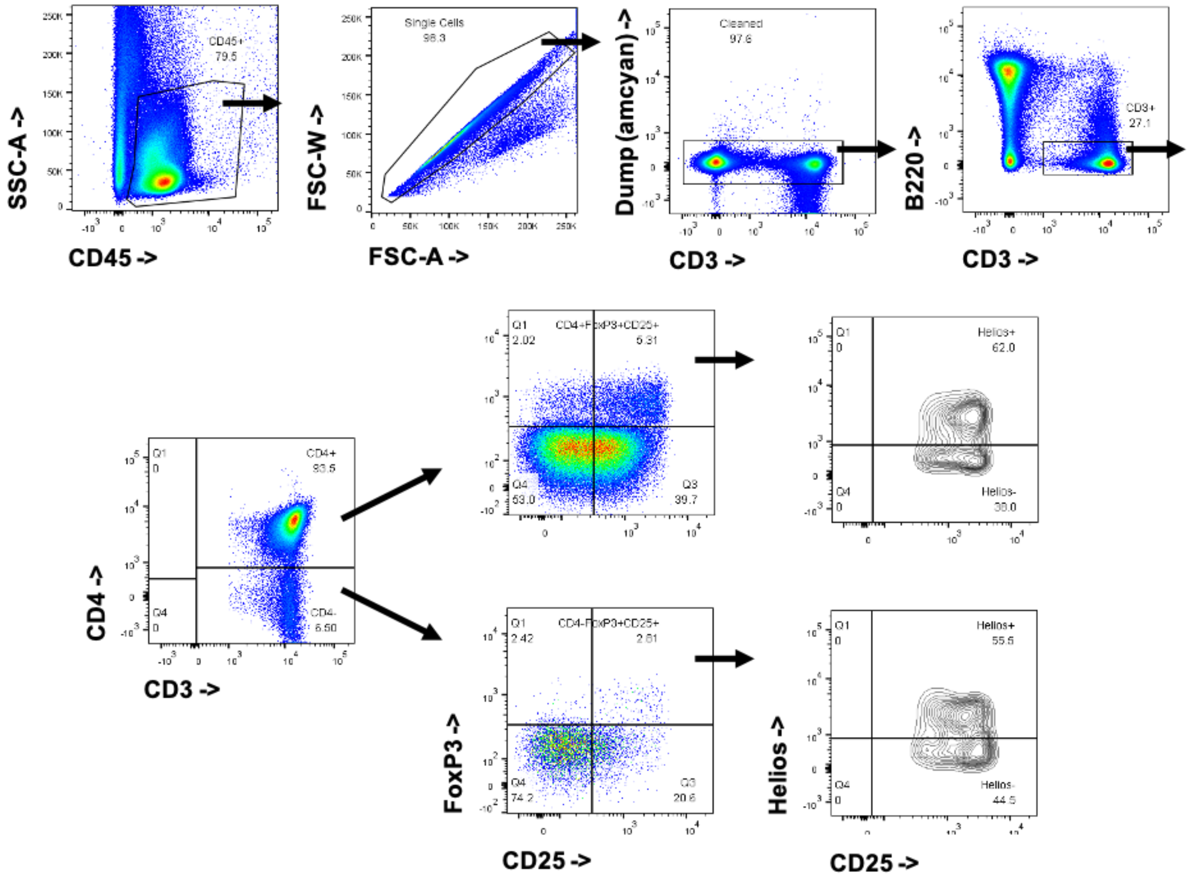

FIGURE S1. Flow cytometry gating strategy. Initially, CD45$^-$ non-lymphocytes, non-single cells, and autoflourescent (amcyan dump) cells were excluded. Next, CD3$^+$B220$^-$ T cells were divided into CD4$^+$ helper T cells and CD4$^-$ cytotoxic T cells. Within both T cell populations, CD25$^+$FoxP3$^+$ double positive regulatory T cells (Tregs) were identified, and further differentiated into Helios+ (natural) and Helios- (induced) Tregs.

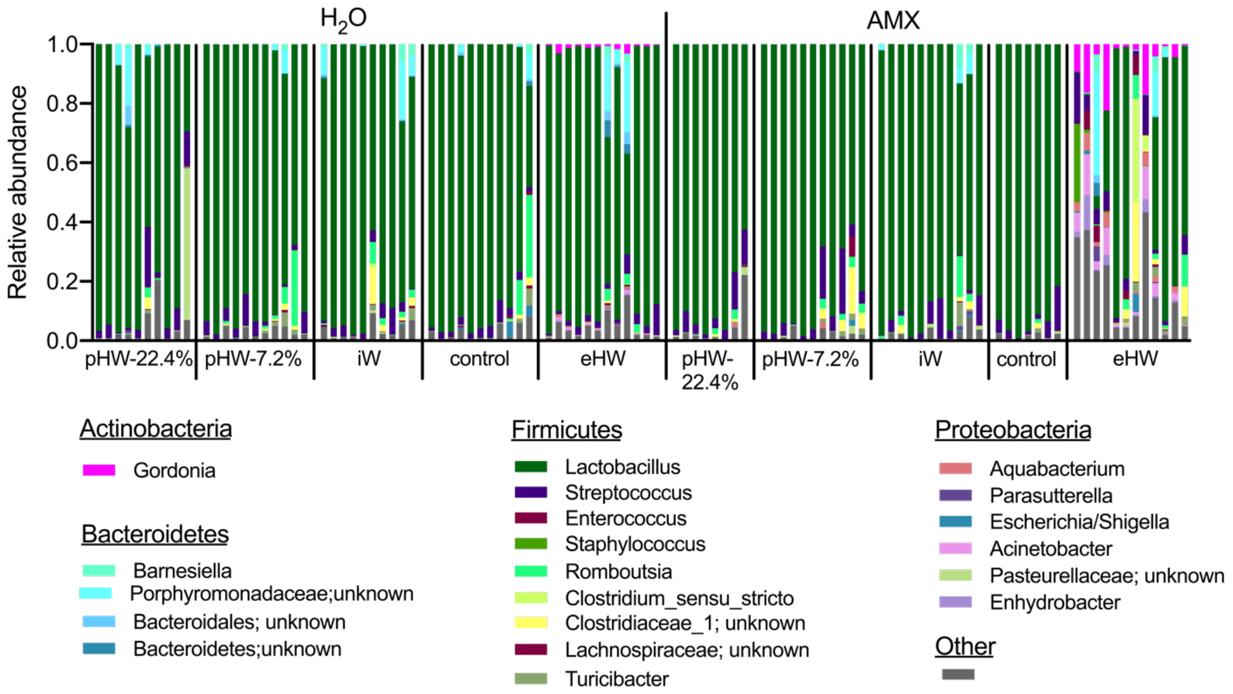

FIGURE S2. Relative abundance of the most abundant bacterial genera in the small intestine of individual rats. Only those genera with a relative abundance of more than 0.05 in at least one rat are shown. The remaining genera are grouped into “Other”.

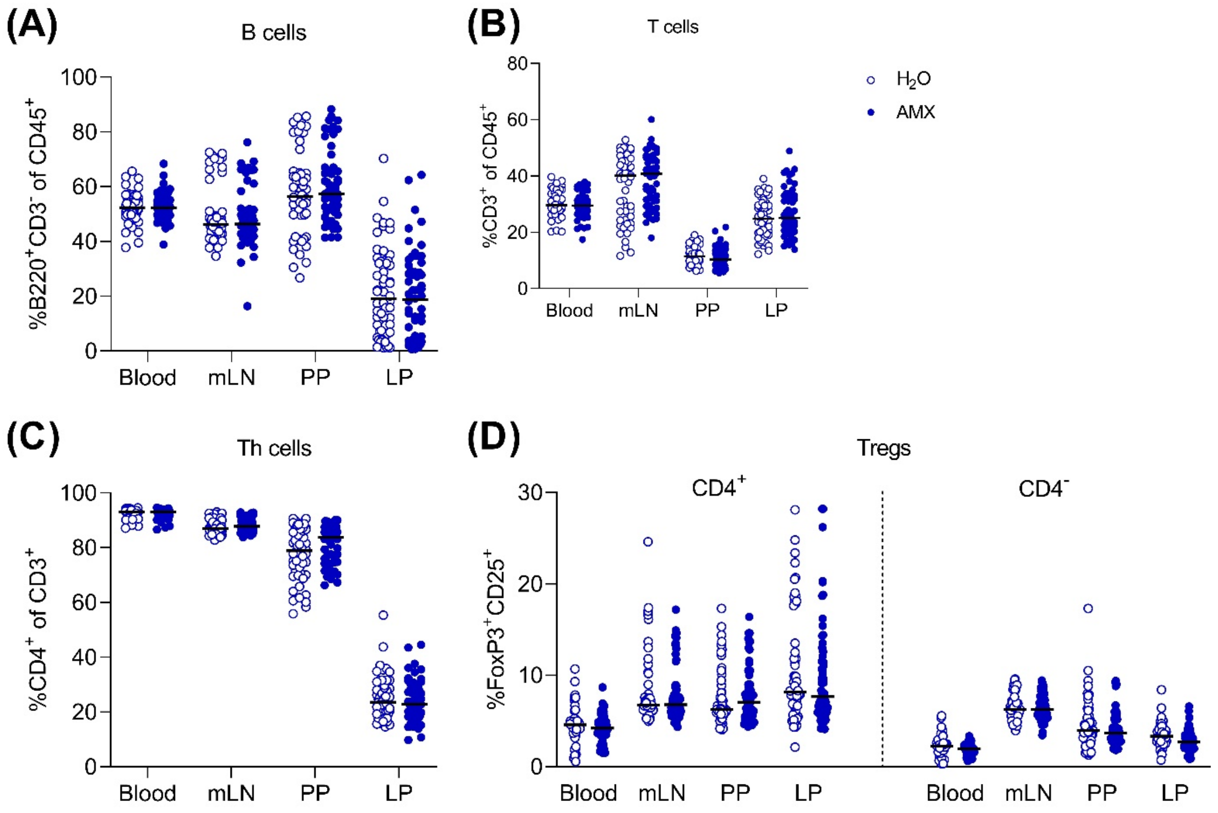

FIGURE S3: Effect of amoxicillin administration on lymphocyte populations. *Total B cells (A), T cells (B), T helper (Th) cells (C) and regulatory T cells (Tregs) (D) in blood, mesenteric lymph nodes (mLN), Peyer's patches (PP) and lamina propria (LP) of conventional (H_2O, open symbols) and amoxicillin (AMX, closed symbols) administered rats. Each symbol represents a single rat and horizontal lines indicate median values.*

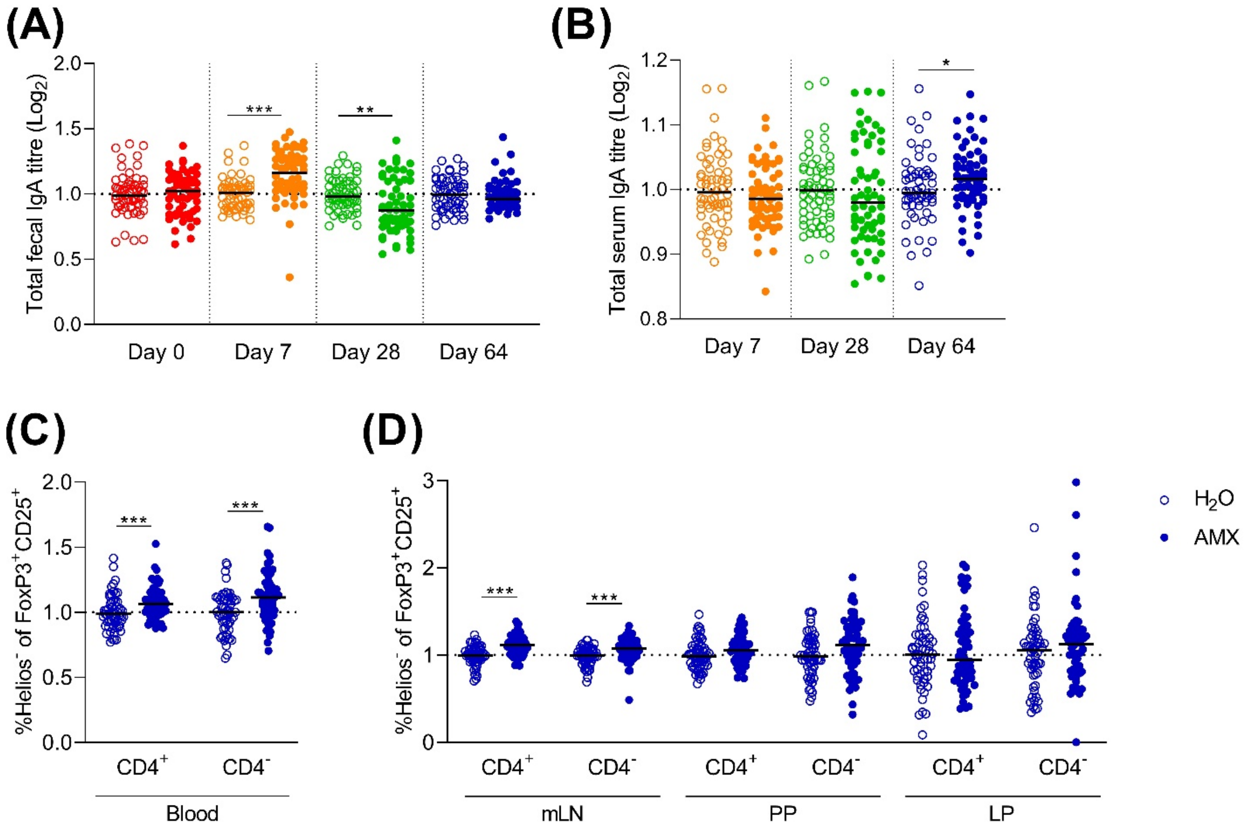

FIGURE S4: Normalised host immune regulation. *Total faecal (A) and total serum (B) IgA titres and fraction of Helios$^-$ Tregs out of the CD25$^+$FoxP3$^+$ Treg populations (both CD4$^+$ and CD4$^-$) in blood (C), and mesenteric lymph nodes (mLN), Peyer's patches (PP) and lamina propria (LP) (D) of conventional (H_2O, open symbols) and amoxicillin (AMX, closed symbols) administered rats. To account for the small variation between the product groups, data are normalised to the median of the water groups of each product group. Each symbol represents a single rat and horizontal lines indicate median values. Statistically significant differences between groups are indicated as *: $p \leq 0.05$, **: $p \leq 0.01$, ***: $p \leq 0.001$.*

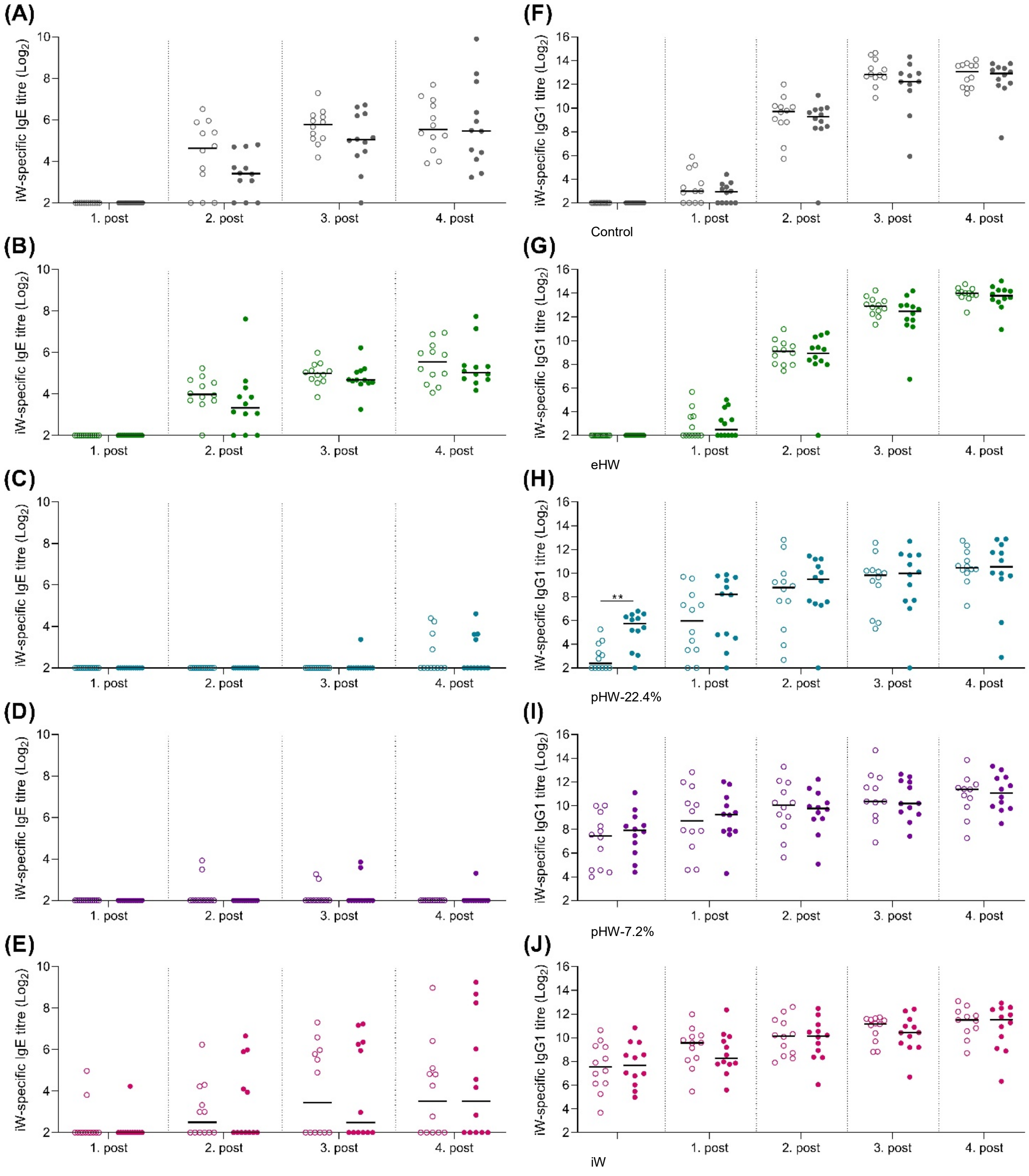

FIGURE S5: Effect of amoxicillin on the primary preventive capacity of the whey protein products. *Intact whey (iW)-specific IgE titres (A-E) and iW-specific IgG1 titres (F-J) in conventional (H_2O, open symbols) and amoxicillin (AMX, closed symbols) rats administered with extensively hydrolysed eHW (B,G), partially hydrolysed pHW-22.4% (C,H) or pHW-7.2% (D,I) or intact (E,J) whey products or water as control (A,F). Each symbol represents a single rat and horizontal lines indicate median values. Statistically significant differences are indicated as **: $p \leq 0.01$.*

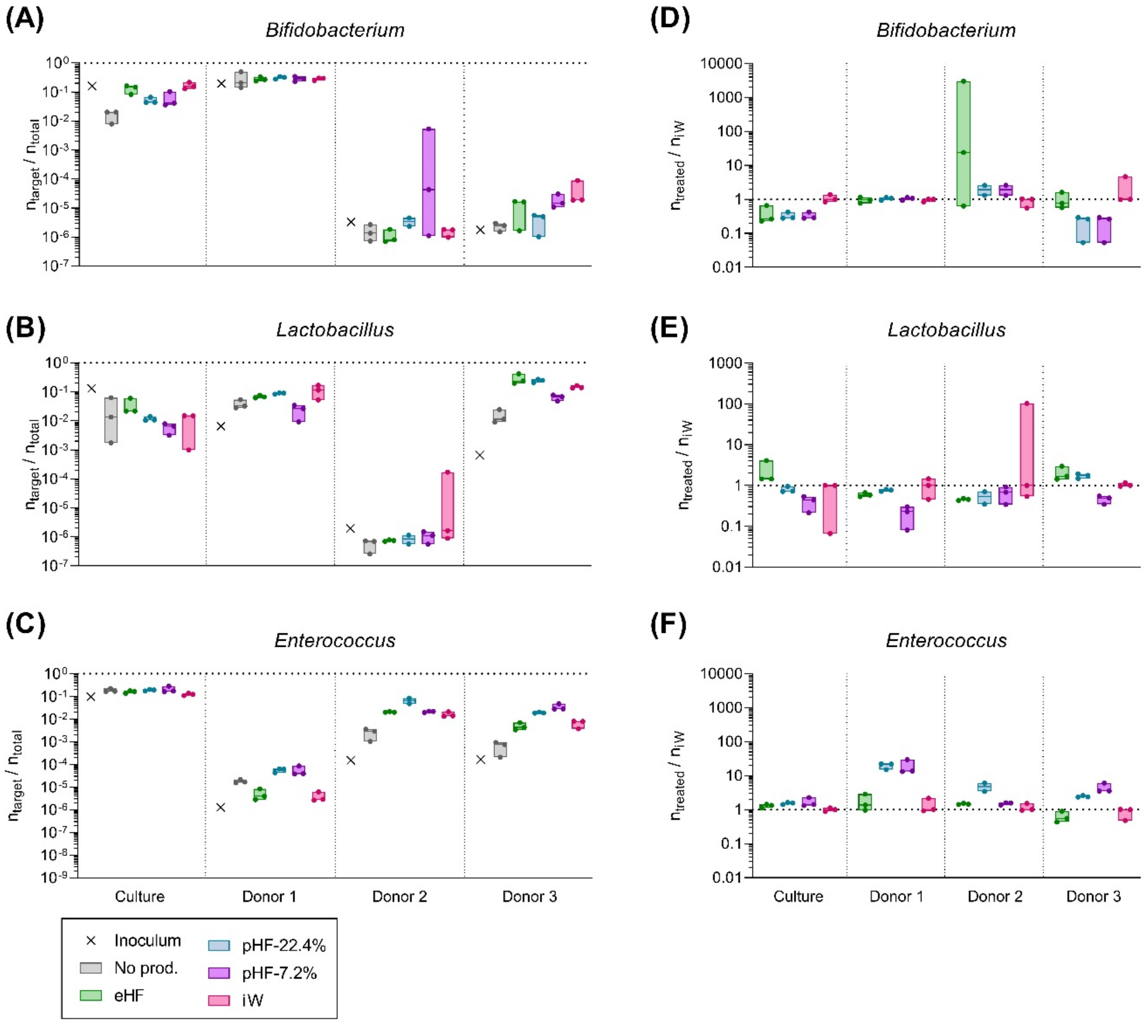

FIGURE S6: Effect of whey protein products on in vitro expansion of bacterial genera derived from healthy infants. *The relative abundance of Bifidobacterium (A, D), Lactobacillus (B, E) and Enterococcus (C, E) after in vitro incubations with extensively (eHW), partially hydrolysed (pHW-22.4% or pHW-7.2%) or intact (iW) whey products. Results for each bacteria is shown relative to the total bacteria (n_{target}/n_{total}) (A-C) and normalised to the median ΔCt of three iW samples ($n_{treated}/n_{iw}$) (D-F).*
